# Modeling the impact of the COVID-19 pandemic on achieving HCV elimination amongst young and unstably housed people who inject drugs in San Francisco

**DOI:** 10.1016/j.drugpo.2024.104452

**Published:** 2024-06-22

**Authors:** Hannah Fraser, Jack Stone, Shelley N Facente, Adelina Artenie, Sheena Patel, Erin C Wilson, Willi McFarland, Kimberly Page, Peter Vickerman, Meghan D Morris

**Affiliations:** a Population Health Sciences, Bristol Medical School, University of Bristol, UK; b School of Public Health, Division of Epidemiology and Biostatistics, University of California Berkeley, Berkeley, USA; c Facente Consulting, Richmond, USA; d Department of Epidemiology and Biostatistics, University of California San Francisco, San Francisco, USA; e San Francisco Department of Public Health, San Francisco, USA; f Department of Internal Medicine, Division of Epidemiology, University of New Mexico, USA

**Keywords:** People who inject drugs (PWID), Young adult people who inject drugs, Unstably housed PWID, Hepatitis C virus elimination, Epidemic modeling

## Abstract

**Background:**

Young adult (18–30 years) people who inject drugs (PWID) face high hepatitis C virus (HCV) prevalence. In San Francisco, where >60% of PWID lack stable housing, barriers hinder HCV treatment access. We assessed progress towards the World Health Organization’s (WHO) HCV elimination goal of an 80% reduction in incidence over 2015–2030, focusing on young (YPWID) and unstably housed PWID in San Francisco.

**Methods:**

We developed a dynamic HCV transmission model among PWID, parameterized and calibrated using bio-behavioural survey datasets from San Francisco. This included 2018 estimates for the antibody-prevalence among PWID (77%) and care cascade estimates for HCV for YPWID (72% aware of their status and 33% ever initiating treatment). Based on programmatic data, we assumed a 53.8% reduction in testing and 40.7% decrease in treatment from 2020 due to the COVID-19 pandemic, which partially rebounded from April 2021 with testing rates then being 31.1% lower than pre-pandemic rates and treatment numbers being 19.5% lower. We simulated different scenarios of how services changed after the pandemic to project whether elimination goals would be met.

**Results:**

Continuing post-pandemic rates of testing and treatment, the model projects an 83.3% (95% credibility interval [95% CrI]:60.6–96.9%) decrease in incidence among PWID over 2015–2030 to 1.5/100pyrs (95% CrI:0.3–4.4) in 2030. The probability of achieving the elimination goal by 2030 is 62.0%. Among YPWID and unstably housed PWID, the probability of achieving the elimination goal by 2030 is 54.8 and 67.6%, respectively. Importantly, further increasing testing and treatment rates to pre-pandemic levels by 2025 only results in a small increase in the probability (67.5%) of the elimination goal being achieved among all PWID by 2030, while increased coverage of medication for opioid use disorder among YPWID and/or housing interventions results in the probability of achieving elimination increasing to over 75%.

**Conclusion:**

The COVID-19 pandemic impeded progress toward achieving HCV elimination. Our findings indicate that existing partial rebounds in HCV testing and treatment may achieve the elimination goal by 2030, with an additional scale-up of interventions aimed at YPWID or unstably housed PWID ensuring San Francisco is likely to achieve elimination by 2030.

## Introduction

The advent of direct-acting antivirals (DAAs) in 2014 marked a breakthrough in the treatment of hepatitis C virus (HCV) infection, providing a well-tolerated, simplified and effective curative therapy (Burstow et al., 2017). In response, the World Health Organization (WHO) set elimination goals, including reducing HCV incidence among people who inject drugs (PWID) by 80% over 2015–2030 (WHO, 2016, 2022). Despite these advancements in treatment, the United States (US) has observed a 124% increase in the incidence of acute HCV in the general population since 2013 (CDC 2020).

In San Francisco, an estimated 2.6% of individuals are HCV antibody positive and PWID constitute three-quarters (73.1%) of these individuals (Facente, Grinstein, Bruhn et al., 2022). Since the mid-2000s in the US, increasing numbers of HCV infections have occurred among young adult PWID under 30 (YPWID) (Abara et al., 2019; Eckhardt et al., 2017; Suryaprasad et al., 2014; Zibbell et al., 2018). In San Francisco, YPWID consistently have high HCV incidence (>20/100pyrs over 2000–2013 (Hahn et al., 2002; Page, Morris, Hahn, Maher & Prins, 2013)) and reduced access to prevention and treatment options, including medication for opioid use disorder (MOUD) which evidence suggests halves the risk of HCV acquisition (Platt et al., 2018). This emphasizes the importance of prioritizing YPWID in HCV elimination efforts (Ganapathi et al., 2019; Krug, Hildebrand & Sun, 2015; Page et al., 2019).

Unstable housing is also high among PWID in San Francisco (61% in 2012 (Coffin, Jin, Huriaux, Mirzazadeh & Raymond, 2015)), far exceeding the global estimate among PWID (25% (Degenhardt et al., 2023)). Unstably housed PWID face an elevated risk of HCV acquisition (Arum et al., 2021), a trend that is pronounced among YPWID, with recent estimates showing unhoused YPWID in San Francisco have 1.5 times greater risk of acquiring HCV than housed YPWID (Morris, Yen, Shiboski, Evans & Page, 2020).

Social marginalization causes PWID to experience heightened barriers to HCV service access (Hall, Le, Majmudar & Mihalopoulos, 2021; Harris & Rhodes, 2013; Risher, Mayer & Beyrer, 2015). Between 2016 and 2020, less than one-tenth of YPWID diagnosed with chronic infection in San Francisco had received treatment (Facente et al., 2021). The COVID-19 pandemic led to further disruptions in HCV services with the shelter-in-place health order in March 2020 decreasing testing and treatment (Facente, Grinstein, Broussard et al., 2022; Hoenigl, Abramovitz, Flores Ortega, Martin & Reau, 2022). Even after COVID-19 protocols were established, PWID and persons experiencing homelessness encountered reduced testing (End Hep C SF, 2024), potentially limiting progress made towards achieving HCV elimination. Survey data from that time also suggested that unstable housing increased among PWID during the pandemic (87.6% by 2022 (Morris et al., 2023)).

Using data on the HCV care cascade among PWID in San Francisco (Facente et al., 2021) as well as the effects of the COVID-19 pandemic on testing and treatment (Facente, Grinstein, Broussard et al., 2022; Hoenigl et al., 2022), we used modelling to evaluate what intervention uptake is needed to achieve the WHO elimination goal of an 80% reduction in HCV incidence among PWID in San Francisco over 2015–2030 (WHO, 2016). We considered different intervention scenarios, with a specific focus on what testing and treatment is needed to achieve elimination among YPWID and unstably housed PWID who have higher HCV incidence and lower levels of service uptake.

## Methods

### Model description

**Fig. 1:**
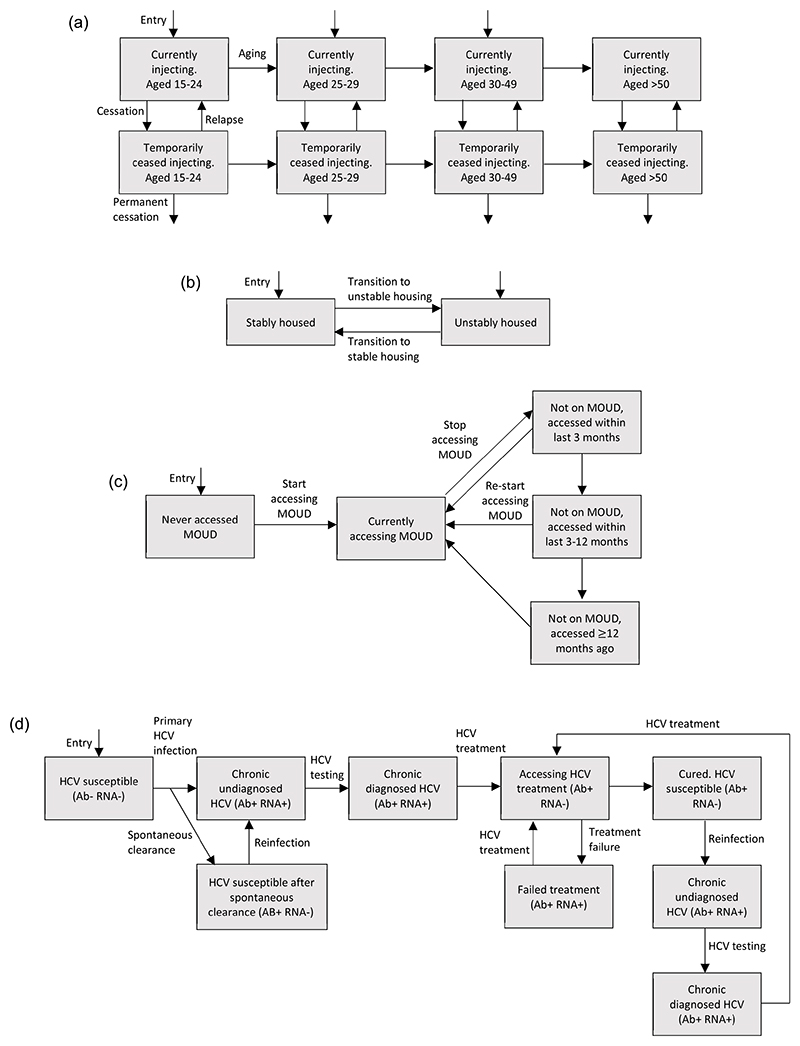
Schematics showing the transitions of PWID between different stratifications. Figure 1a: Schematic showing the transitions of PWID between different age and injecting states. A PWID in any state can also be in any intervention state, housing state, and infection state. Note that background mortality is not shown on the figure for simplicity. Figure 1b: Schematic showing the transitions of PWID between different housing states. A PWID in any state can also be in any MOUD state, age stratification, injecting state, and infection state. Note that entry is into either housing state. Background mortality and permanent cessation are not shown on the figure for simplicity. Figure 1c: Schematic showing the transitions of PWID between different MOUD states. A PWID in any state can also be in any housing state, age stratification, injecting state, and infection state. Note that PWID enter the model never having accessed MOUD. Background mortality and permanent cessation are not shown on the figure for simplicity. Figure 1d: Schematic showing the transitions of PWID between infection states. A PWID in any state can also be in any housing state, age stratification, injecting state, and MOUD state. Note that all PWID enter as susceptible to HCV. Spontaneous clearance associated with re-infection, background mortality and permanent cessation are not shown for clarity.

We adapted an existing dynamic HCV transmission model among PWID for San Francisco (Fraser et al., 2019). The modelled PWID population was stratified by age (in years, 18–24, 25–29, 30–49 and ≥50), injecting status (currently injecting or temporarily ceased), housing status (currently unstably housed or stably housed), intervention status (never accessed MOUD, currently on MOUD, recently accessed (but not currently) MOUD either in past 3 or 12 months, and accessed MOUD >1 year ago) and HCV-infection status (Fig. 1). The model incorporated a time-varying rate of initiating injecting, with PWID leaving the model due to mortality (background and drug-related). Currently injecting PWID can temporarily cease injecting (rate dependent on MOUD status and age), and can then relapse (age dependent rate) back to currently injecting or permanently cease injecting and leave the model (unaffected by MOUD status).

Upon initiating injection drug use, individuals enter the model into the first three age categories (15–24, 25–29, 30–49), as stably or unstably housed, and not accessing MOUD (Fig. 1). The proportion entering each age category and housing status varies over time. Individuals transition through the age groups. We assume the recruitment rate of PWID decreases over time to fit to the aging PWID population seen in San Francisco with this decrease occurring 20–40 years ago. PWID transition between unstable and stable housing, with this movement varying over time (Morris et al., 2020).

HCV transmission occurs at a rate dependent on the prevalence of chronic HCV infection, with transmission risk being reduced if PWID are on MOUD (Platt et al., 2018), but increased if they are currently unstably housed (Morris et al., 2020) or are YPWID. Mixing between PWID to form transmission contacts ranges from random to partially like-with-like, either by age or housing status (Fraser et al., 2019). PWID enter the model susceptible to HCV. Once infected, some spontaneously clear their infection (Grebely et al., 2014; Micallef, Kaldor & Dore, 2006) and become susceptible again (antibody positive, RNA negative), whilst all others develop chronic infection which are initially undiagnosed (antibody positive, RNA positive) (Fig. 1d). Chronically infected PWID can undergo screening, and once diagnosed can initiate treatment. A proportion of those treated achieve a sustained virological response (SVR – cure) and transition to the cured group after on average 12 weeks. Those who do not achieve SVR transition to the treatment failure group, and can be retreated at the same rate as treatment naïve individuals. Cured individuals can become reinfected, following which they can be screened and initiated onto treatment at the same age-dependent rate as for primary infection.

### Model parameterisation and calibration

The model was parameterised with data from: the UFO Study, a longitudinal study among YPWID (aged <30 years) in San Francisco over 1997–2018 (Hahn et al., 2002; Morris et al., 2020; Page et al., 2009); the National HIV Behavioral Surveillance (NHBS) System for PWID over 2009–2018, a cross-sectional survey run every 3–4 years across multiple US cities including San Francisco (CDC, 2012, 2015, 2020; Coffin et al., 2015; Kral et al., 2010); and the Urban Health Study (UHS), a cross-sectional survey from inner-city San Francisco with data used from 1998 to 2000(Tseng et al., 2007). Further details of these studies are given in Supplementary Table 1, with model parameters and their sources given in Table 2.

Estimates of the age when individuals initiate injecting were obtained from UFO, UHS and NHBS data, with a greater proportion of older individuals initiating injecting over time (Table 1). The temporary cessation rate and relapse rate for PWID aged <30 years came from UFO data (Evans, Hahn, Lum, Stein & Page, 2009). The relapse rate for PWID aged ≥30 years also came from UFO data for those aged ≥27 (Evans et al., 2009) as there was no data specifically for those aged ≥30. The temporary cessation rate for PWID aged 30–49 years and ≥50 years, and the number of individuals initiating injecting annually were estimated through model calibration to the estimated number of YPWID, overall number of PWID and the percentage of PWID aged ≥30 years that are 30–49 years (CDC, 2020; Facente et al., 2018, 2021).

Time-varying recruitment rates onto MOUD were estimated through calibrating to data on the coverage of MOUD at different time points. Among YPWID, we assumed that MOUD initiated in 2000 and increased from 2.6% to 12.2% over 2006 to 2015. MOUD was assumed to have higher coverage among PWID in older age groups, with estimated coverage levels of 46.1% and 44.4% in 2018 among those aged 30–49 and ≥50 years, respectively. MOUD coverage did not differ by unstable housing status.

Based on UFO and NHBS data, we assumed the proportion of PWID unstably housed increased linearly from 55.9% to 73.8% over 2009 to 2018 ((Coffin et al., 2015) and analysis for this project), with no difference across age groups. The transition rate from unstable to stable housing was initially estimated from UFO data. However, initial model calibrations suggested that the rate needed to be lower to reproduce the high prevalence of unstable housing among PWID, and so the uncertainty range was extended. The transition rates back to unstable housing were estimated through model calibration to the proportion unstably housed over time.

We estimated age-specific HCV testing and treatment rates through model calibration to the care cascade from the 2018 NHBS survey. We assumed treatment started in 2016, and the proportion ever HCV treated among those diagnosed increased over time to a lower level in YPWID (33.3%) than older PWID by 2018 (51.6% of PWID aged 30–49 and 60.4% of PWID aged ≥50). Analyses of NHBS data suggested no difference in levels of testing and treatment for those accessing MOUD (versus not accessing MOUD). However, among unstably housed PWID, there was a 19% reduction in testing and 34% reduction in ever treatment compared to those stably housed.

The model was calibrated using an approximate Bayesian computation Sequential Monte Carlo (ABC SMC) method to calculate summary statistics up to 2018 on: population size estimates for different age groups; proportion of PWID of different ages accessing MOUD for different years; proportion of PWID unstably housed; HCV incidence among YPWID (up to 2013); and the care cascade among PWID of different ages (Table 2 and Supplementary Materials for further details). Through this calibration, we estimated baseline transmission rates (among stably housed PWID not accessing MOUD) for PWID aged <30 and ≥30. The final set of 5000 parameters from the ABC SMC were defined as the initial model fits. Estimates of HCV sero-prevalence among PWID aged <30 years in 2018 were used for model validation as was the estimated HCV incidence among YPWID for 2013–2018.

Further information on the modelling can be found in the Supplementary Materials.

### Model analyses

The baseline model (denoted as the status quo (SQ) model) fits were used to estimate the decrease in HCV incidence achieved over 2015–2030, incorporating the effect of the COVID-19 pandemic on decreasing rates of testing and treatment and increasing rates of unstable housing. This included a 53.8% decrease in community-based anti-HCV testing rates and a 40.7% decrease in treatment numbers from 2020 in San Francisco, which partially rebounded from April 2021 with testing rates being 31.1% lower than pre-pandemic rates and treatment numbers being 19.5% lower (End Hep C SF, 2024). Actual treatment rates amongst PWID diagnosed with HCV for 2020 and from April 2021 were calibrated to give these changes in treatment numbers. Unstable housing among PWID increased to 87.6% by June 2022 (Morris et al., 2023). We assessed whether the WHO HCV elimination goal of an 80% reduction in incidence over 2015–2030 (denoted the ‘elimination goal’) could be met with this increase in unstable housing and the partial rebound in HCV testing and treatment numbers that occurred in 2021 in San Francisco. Model projections were continued to 2050 to estimate when the elimination goal would be achieved.

We then modelled the likelihood of achieving the elimination goal for eight alternative scenarios incorporating potential improvements in HCV testing and treatment services, MOUD coverage and/or housing provision that could be introduced after 2023 for different PWID groups (all PWID, YPWID, or unstably housed PWID). Broadly, these scenarios considered the impact of reversing the detrimental effects of the COVID-19 pandemic on decreasing testing rates and treatment numbers or increasing levels of unstable housing, and correcting for lower levels of service access among YPWID and unstably housed PWID.

The eight scenarios are:

- Scenario 1: COVID-19 pandemic did not occur. Counterfactual of no change in testing and treatment due to the pandemic from March 2020.
- Scenario 2: Rebound in testing and treatment in all PWID. Testing rates return to pre-pandemic levels by 2025 among all PWID, as does treatment rates if post-pandemic rate is below pre-pandemic rate (linear increase over 2024–2025 from level seen after the pandemic). If post-pandemic treatment rate is above pre-pandemic rate, then treatment rate does not change;
- Scenario 3: MOUD increases in YPWID over 2024–2025 (from 25.5% (14.3–39.6%) accessing MOUD in last year to 46.1% (39.3–53.0%); same as among PWID aged 30–49) and sustained thereafter;
- Scenario 4: Rebound in testing and treatment in all PWID and MOUD increases in YPWID. Scenario 2 plus Scenario 3;
- Scenario 5: Increased housing among all PWID. Unstable housing levels decrease linearly over 2024–2025 among all PWID (from 87.6% to pre-pandemic level of 73.8% [95% CrI: 69.5–77.8%])
- Scenario 6: Increased housing in all PWID (scenario 5) plus increase in HCV testing and treatment levels among unstably housed PWID to same level as stably housed PWID by 2024.
- Scenario 7: Rebound in testing and treatment and increased housing in all PWID. Scenario 2 plus Scenario 5; and
- Scenario 8: Rebound in testing and treatment in all PWID and increased housing in all PWID plus increase in HCV testing and treatment levels among unstably housed PWID. Scenario 2 plus Scenario 6.

Model results are provided separately for PWID, YPWID and unstably housed PWID to assess whether YPWID and unstably housed PWID may need additional interventions to achieve the elimination goals.

### Uncertainty analyses

To ascertain which parameters were important for determining variability in our projections of the decrease in HCV incidence achieved over 2015–2030 for the status quo scenario, a linear regression analysis of covariance (ANCOVA) was performed on our 5000 model fits (Briggs, Claxton & Sculpher, 2006). The proportion of the sum of squares contributed by each parameter was calculated to determine each parameters’ importance to the variability in our projections.

## Results

**Fig. 2:**
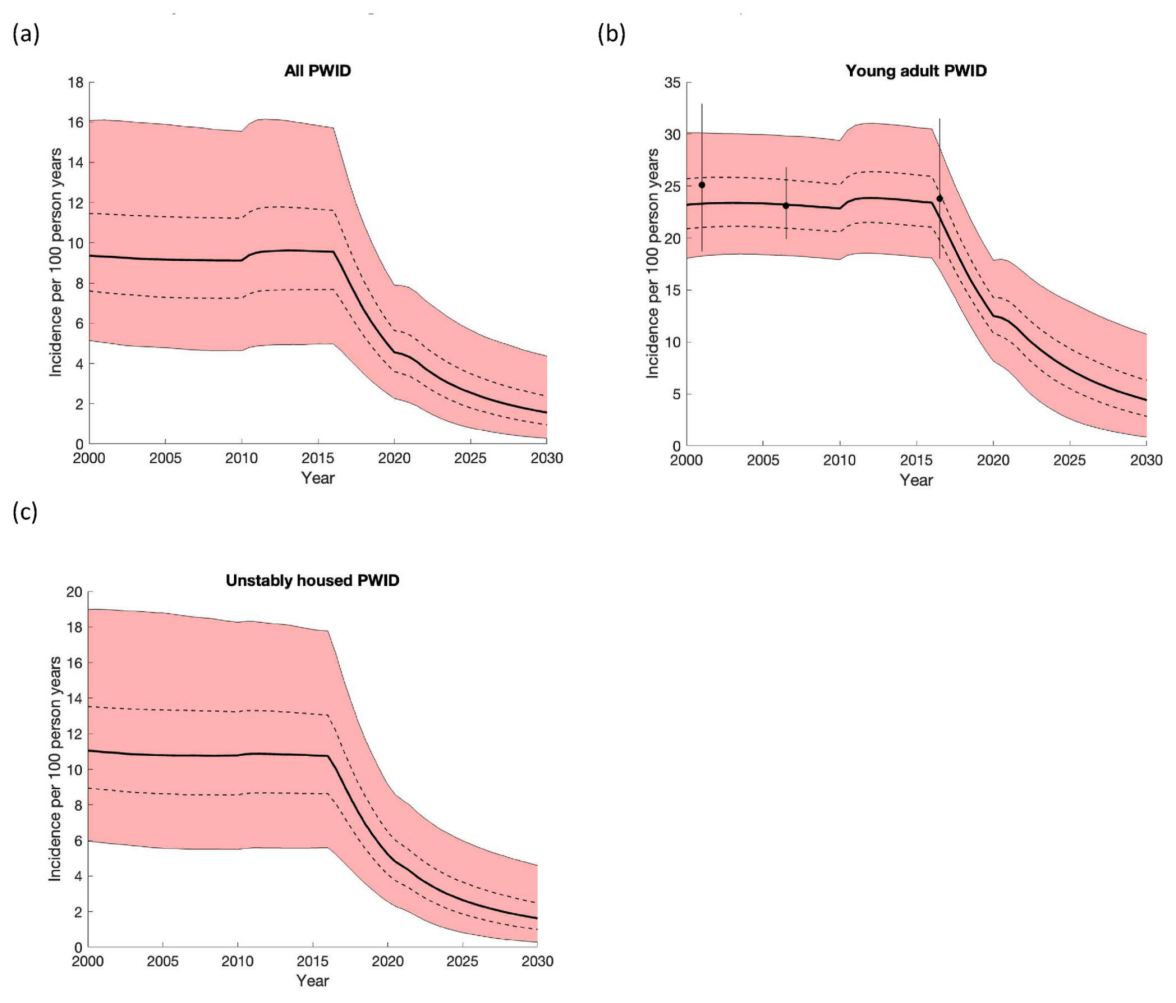
The projected incidence among (a) all people who inject drugs (PWID), (b) young adult people who inject drugs (YPWID) in San Francisco, and (c) unstably housed PWID. MOUD is assumed to increase from 2000 over the time period, with HCV testing and treatment starting in 2016. The thick black line shows the median of the model runs while the red area shows the 95% credibility intervals of the 5000 baseline model fits and the dashed lines give the interquartile range. The black points and whiskers give the mean and 95% confidence interval of incidence data among YPWID in San Francisco; the 2015.5 data point is not calibrated to.

A comparison of the model with available data used for model calibration (Table 2) is shown in Fig. 2 (HCV incidence) and Supplementary figure 1. These figures illustrate that the model generally agreed well with available data and shows how HCV diagnosis levels and HCV treatment levels increased sharply after 2015.

### Epidemic projections and scenario analysis

#### All PWID

**Fig. 3:**
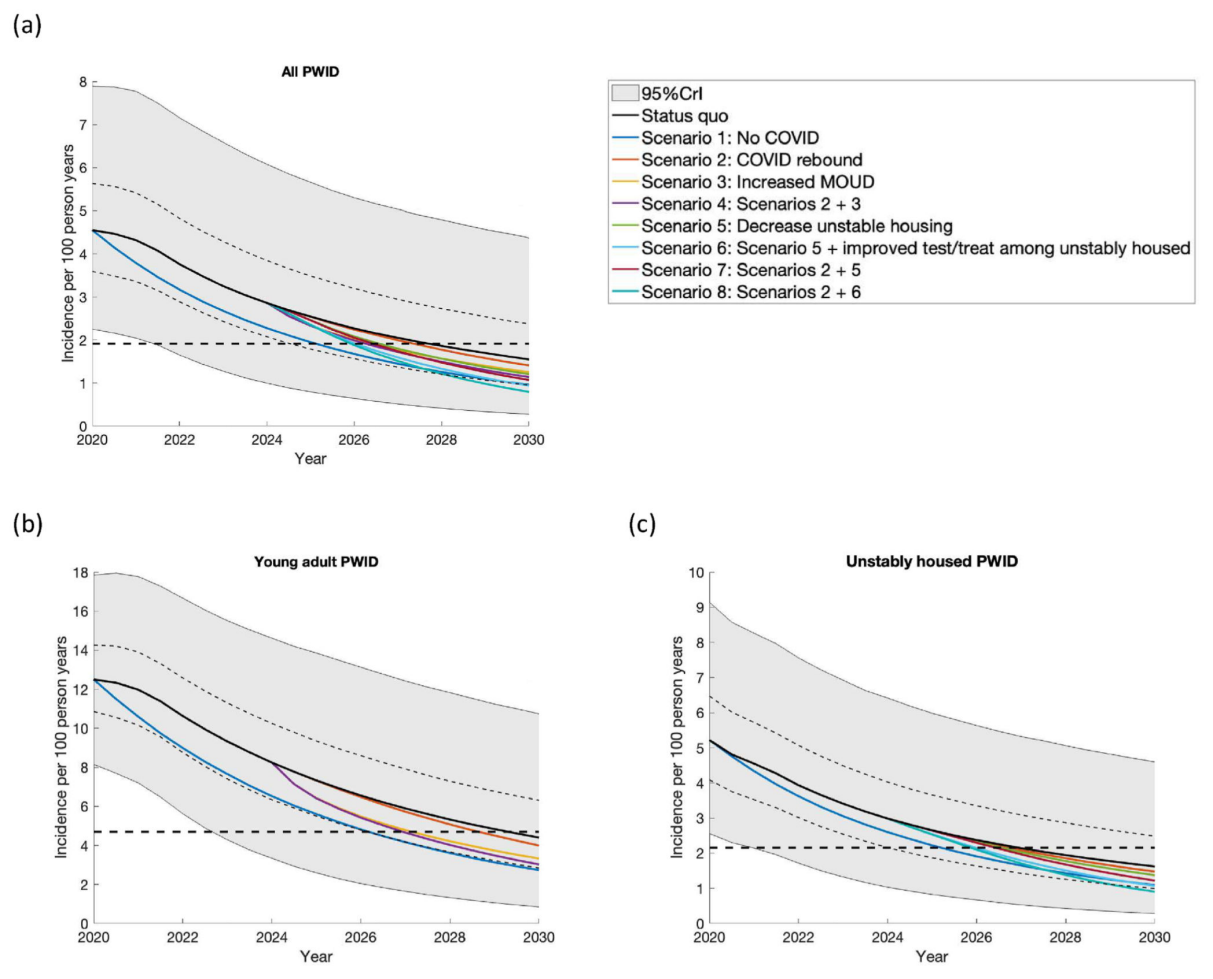
The projected impact of different intervention scenarios from 2024 to 2030 on HCV incidence among (a) all PWID, (b) YPWID aged < 30 years and (c) unstably housed PWID. MOUD is assumed to start in 2000. Incidence is estimated among susceptible PWID. Each panel shows the median of the baseline model fits and their interquartile range and 95% credible interval (solid black line, thin dashed lines and shaded grey area) and different scenarios related to the specific subgroups. The black dashed line in each figure represents the WHO elimination goal of an 80% reduction in the median modelled HCV incidence since 2015. *Scenarios are:* *Status* *quo:* Continuing with testing and treatment as during COVID; Scenario 1: Counterfactual of no change in testing and treatment over March 2020 to present; Scenario 2: Rebound in the testing and treatment levels to pre-COVID levels by 2025 among all PWID (reversing the 59.1% decrease that was seen due to COVID); Scenario 3: Increase in MOUD levels in 2024 among YPWID (from 25.5% (14.3–39.6%) accessing MOUD in the last 12 months to 46.1% (39.3–53.0%), the same as among PWID aged 30–49); Scenario 4: Scenario 2 plus Scenario 3; Scenario 5: Decrease in unstable housing levels by 2025 among all PWID (from 87.5% to 73.8% (69.5–77.8%)); Scenario 6: Scenario 5 plus increase HCV testing and treatment levels in 2024 among unhoused PWID; Scenario 7: Scenario 2 plus Scenario 5; and Scenario 8: Scenario 2 plus Scenario 5 plus Scenario 6.

In agreement with data, the model projects HCV incidence in San Francisco was fairly stable until 2016 (Fig. 2), with only a slight reduction due to the introduction of MOUD in 2006. With the introduction of DAAs in 2016 incidence quickly decreases, slowing in 2022 due to decreases in testing and treatment during the COVID-19 pandemic. Under the SQ scenario, the model projects a 83.3% (95% credibility interval [95% CrI]: 60.6–96.9%) decrease in HCV incidence over 2015–2030 from 9.6/100 pyrs (95% CrI: 5.0–15.9) in 2015 to 1.5/100pyrs (95% CrI: 0.3–4.4) in 2030. The probability of achieving the elimination goal of an 80% decrease in incidence over 2015–2030 is 62.0% (3100/5000 model runs). For this scenario, we estimate that a median of 12,421 (95% CrI:7,017–23,920) treatments are needed among all PWID over 2015–2030, with 1,618 (95% CrI: 795–3,156) treatments being needed over 2025–2030. This translates to 23.6% (95% CrI: 14.9–36.2) of undiagnosed PWID needing to be tested each year and 27.0% (95% CrI: 12.2–48.2) of those diagnosed needing to be treated each year over 2025–2030. Conversely, if decreases in testing and treatment had not occurred during the pandemic (Scenario 1), HCV incidence would have decreased by 89.5% (95% CrI: 78.5–97.2%) over 2015–2030 (Fig. 3).

Table 3 summarises the projections for the eight modelled scenarios for each PWID subgroup including additional projections for the median year when elimination will occur. Under scenario 2, where testing and treatment rates return to pre-pandemic (2019) levels by 2025, the model projects a small increase in the probability (from 62.0 to 67.5%) of achieving the elimination goal by 2030 when compared to the status quo scenario. Alternatively, under the other scenarios where the return in testing and treatment to pre-pandemic levels is paired with other improvements (scenarios 4, 7 and 8), the probability of reaching the elimination goal by 2030 increases considerably to over 80% (Table 3). For example, combining rebounds in testing and treatment with decreases in unstable housing and increases in testing and treatment among unstably housed PWID (Scenario 8), results in a 95.6% probability of the elimination goal being reached by 2030 (Table 3). This modelled effect is primarily due to the impact of decreasing unstable housing and increasing testing and treatment among unstably housed PWID, which by itself (when added to status quo scenario) results in a 92.4% probability of reaching the elimination goal by 2030 (see scenario 6 in Table 3).

#### YPWID

Under the SQ scenario, the model projects a similar decrease in HCV incidence among YPWID as among all PWID over 2015–2030 (83.3% vs 81.3% (95% CrI: 57.0–96.2%) among YPWID) and so the probability of achieving the HCV incidence elimination goal among YPWID by 2030 is similar at 54.8% (2,739/5,000 model runs). As for all PWID, returning testing and treatment rates to pre-pandemic levels (Scenario 2) results in a small increase in the probability of achieving the elimination goal by 2030 (60.9% or 3,046/5,000 model runs), while it increases dramatically (to 77.6%) if it is combined with improved access to MOUD among YPWID (Scenario 4).

### Unstably housed PWID

In the SQ scenario, the decrease in incidence among unstably housed PWID is 84.5% (95% CrI: 63.5–97.1%) over 2015–2030 with there being a 67.6% probability that the elimination goal will be reached. As for other groups, fully returning testing and treatment rates to pre-pandemic levels only marginally increases the probability that the elimination goal will be reached by 2030 (with 72.8% probability, Scenario 2). In contrast, additional reductions in unstable housing and increases in testing among unstably housed PWID dramatically increases the probability of achieving the elimination goal by 2030 to 95.3% (Scenario 8).

### Sensitivity analysis

Our ANCOVA analysis indicates that uncertainty in the risk ratios associated with reductions in treatment and diagnosis rates among unstably housed PWID contribute most to variability in the decrease in incidence over 2015–2030 for the SQ model, contributing 48.3% and 16.8% of the variation respectively. Uncertainty in the treatment rates for those aged 30–49 and ≥50 contribute a further 6.4% and 5.0%, respectively, with all other parameters contributing <5% towards the variability.

## Discussion

The impact of COVID-19 on reducing HCV testing and treatment provision among PWID in San Francisco had implications for realizing the WHO’s HCV elimination goal of an 80% reduction in HCV incidence over 2015–2030 in this population. The pre-pandemic trajectory in San Francisco would have achieved the elimination goal among PWID an estimated 4.5 years ahead of the 2030 goal. Although it is still fairly likely (62.0% probability) that the elimination goal will be reached by 2030, the reduced levels of testing and treatment that occurred during and after the pandemic has delayed the chance of reaching elimination by an estimated 3 years and made the timeframe uncertain. The likelihood of achieving the elimination goal improves slightly (67.5% probability) with a full return to pre-pandemic levels of testing and treatment services by 2025. Conversely, it improves further (>75% probability) with an expansion in MOUD access for YPWID and improves considerably (>90% probability) with a reduction in unstable housing among PWID paired with an increase in testing and treatment rates among unstably housed PWID (to same level as other PWID).

Lastly, despite lower levels of testing and treatment rates and MOUD coverage among YPWID and unstably housed PWID, our findings suggest there are only small differences (of about 1 year) in when the elimination goal will be achieved among these PWID subgroups compared to other PWID. Although this is encouraging, it should not detract from the importance of directing interventions to these subgroups to ensure comprehensive elimination because our results suggest they have large impact. Opportunities exist to co-locate HCV testing and treatment services within overdose services as the city expands its response to the overdose epidemic with funds from a large city settlement (City Attorney of San Francisco, 2023).

### Strengths and limitations

The strength of our modelling is undertaking a detailed analysis of a site-specific model for San Francisco. We leveraged care cascade estimates generated from original research data and robust programmatic data to develop a comprehensive model reflecting the current landscape of HCV testing, treatment and epidemiology in San Francisco, resulting in improved model precision compared to prior research (Fraser et al., 2019). A particular strength is our use of programmatic data on levels of community-based HCV testing and treatment from San Francisco over 2019–2023 (End Hep C SF, 2024) for parameterising how the COVID-19 pandemic reduced rates of testing and treatment, and how that rebounded in subsequent years.

Despite this, our analyses had certain limitations. Firstly, we used point estimates for the reduction in testing and treatment during and following the COVID-19 pandemic based on programmatic data (End Hep C SF, 2024). No uncertainty was included because they were not sample estimates. We also assumed this reduction and any rebound in testing and treatment after the COVID-19 pandemic occurred equally across all sub-groups of PWID. Although this is uncertain, there was no available data to suggest otherwise. We do not think this limitation will have affected our projections majorly as shown by the small difference in our projections due to existing differences between YPWID, unstably housed PWID and other PWID. We also did not have data on how MOUD or syringe service provision (SSP) may have changed during the COVID-19 pandemic, although social distancing measures may have limited access. Data from elsewhere in the US suggest that MOUD access may have increased or decreased, while SSP access is likely to have decreased (Aponte-Melendez et al., 2021; Feder et al., 2022; Kawasaki, Zimmerman, Shen & Zgierska, 2023; Taylor, Cantor, Bradford, Simon & Stein, 2023). This adds uncertainty to our projections and emphasizes the importance of continuously monitoring intervention outputs to better understand how provision is changing.

Local estimates of the testing and treatment rates pre-pandemic were based on self-reported data from PWID because programmatic data on HCV testing and treatment was not just for PWID and did not include all providers. As uncertainty in the risk ratios associated with testing and treatment rates for those unstably housed, and the treatment rates among those aged >30 years contributed most to the variability in the decrease in incidence over 2015–2030, these rates are important factors to understand. Ensuring that all testing and treatment by clinics and health services is tracked and includes a persons’ injecting history is essential for accurately understanding the pathway of HCV care and for simulating its impact. Alongside this, tracking all negative tests under-taken and stratifying testing and treatment estimates by subgroups (e.g., PWID, YPWID and unstably housed PWID) in surveillance systems is important to improve the usability of this data, and helps ensure that no group is left behind in HCV elimination efforts. San Francisco is now collecting and analysing negative tests as part of their city’s sentinel surveillance programme for HCV, which future models will incorporate.

Second, the absence of incidence data for older PWID (>30 years of age) meant we had to rely on prevalence estimates for calibrating that aspect of the model. Although our prevalence estimates were reasonably recent (2018) using robust local programmatic and research data sources (Facente et al., 2021), the lack of incidence estimates for older PWID including those with unstable housing meant the modelled incidence projections were sometimes uncertain. Despite this, the availability of incidence data from the UFO study (Hahn et al., 2002; Page et al., 2013), a well-established cohort of YPWID, allowed us to calibrate our model to incidence estimates at multiple time points, and the cohort also allowed us to incorporate differences in MOUD coverage and mixing patterns among different ages. The UFO study also allowed us to estimate the degree to which young unstably housed PWID have higher HCV incidence. Although this was incorporated into the model, we did not assess or include the underlying mechanism by which that may occur, so hindering the development of specific interventions to reduce this risk. Unfortunately, enrolment for the UFO study ended in December 2016, and so available data used to calibrate the model was generally from before 2018. This prevented us from validating our model projections against prevalence or incidence data for more recent years. Despite this, our model projections suggesting a decrease in incidence (59.3% [95% CrI: 41.1–78.8] decrease over 2015–2022) do align with case report data for San Francisco over 2015–2022 indicating a 60% reduction in annual reported HCV chronic cases (personal communication San Francisco Public Health Department). However, it is hard to interpret this data.

Third, our definition of MOUD encompasses methadone and buprenorphine use, and the duration on MOUD is based on a systematic review (Bao et al., 2009). Unfortunately, this review and our model did not account for the differences in how methadone may be accessed, such as office-based MOUD. Future modelling should account for this and incorporate potential longer durations accessing MOUD. Further, while data suggest an increase in drug-related mortality rates since the emergence of fentanyl, and a possible further increase during the COVID-19 pandemic (Appa et al., 2021; City & County of San Francisco, 2023), our models did not incorporate such variability in mortality rates because preliminary modelling (see ANCOVA analysis) and previous analyses have shown that such variations do not affect the impact of HCV treatment on transmission (Martin et al., 2011).

### Implications

The *End Hep C SF* initiative was the first in the USA to develop a city-focused plan to eliminate HCV. Under this initiative, an upsurge in HCV testing and treatment occurred, while the wealth of data in San Francisco has enabled a comprehensive exploration of the care cascade among PWID and its potential impact. Ours is the first modelling study to also include housing interventions, including improving testing and treatment among those unstably housed alongside modelling the impact of reducing unstable housing. Moreover, this study is novel as it assessed elimination progress among YPWID and unstably housed PWID.

On a global scale, the COVID-19 pandemic introduced obstacles and slowdowns in HCV elimination agendas, jeopardizing the realization of the WHO’s HCV elimination goals (Blach et al., 2021). However, in line with our previous modelling analysis among MSM in San Francisco (Artenie et al., 2023), we project that elimination may still be achieved by 2030 despite reductions in testing and treatment due to the COVID-19 pandemic. This is due to a partial return in testing rates and treatment numbers from April 2021, with an additional scale-up of interventions aimed at YPWID or unstably housed PWID being particularly important for ensuring that San Francisco is back on course to achieve these goals. While other studies have examined the pandemic’s impact on HCV disease burden and coverage of interventions (Aponte-Melendez et al., 2021; Feder et al., 2022; Kawasaki et al., 2023; Taylor et al., 2023), few have considered how services have resumed afterwards.

As the US Congress considers the proposed budget to support a national plan to eliminate HCV, our study presents timely evidence of the value of sustained investments in PWID health to safeguard progress and achieve elimination by 2030. Our results show the feasibility of achieving the WHO’s elimination goal of an 80% reduction in HCV incidence over 2015–2030 in San Francisco through a partial return in testing and treatment rates disrupted by the pandemic. Indeed, a well-defined strategy that also enhances access to housing and MOUD – especially among YPWID and unstably housed PWID – is particularly important to ensure elimination is achieved while ensuring equitable treatment and service availability for those most in need.

## Supplementary Material

Supplementary file

## Figures and Tables

**Table 1:** Model parameters with uncertainty bounds (prior distributions) and posterior distributions from the model calibration. For normal and log-normal distributions, the prior range gives the mean and 95% CI.

Parameter	Prior range	Distribution	Posterior range	Notes/References
*PWID demographic and injecting related parameters*				
Percentage of PWID that initiate injecting aged 15-24 in 2010	75.9% (71.2–80.1%)	Normal	74.7 (71.5–79.3)	Estimated through calibration to data on age of first injecting from UHS (Kral et al., 2010) (2010 data) and NHBS (CDC, 2020) (2018 data).
Percentage of PWID that initiate injecting aged 15-24 in 2018	67.8% (63.3–72.1%)	Normal	68.1 (64.2–71.4)	
Percentage of PWID that initiate injecting aged 30–49 in 2010	11.7% (8.6–15.3%)	Normal	11.3 (8.9–14.7)	Percentage of PWID initiating injecting aged 25–29 calculated using 100 -% initiated aged 15–24 -% initiated age 30–49. Linear change between 2010 and 2018.
Percentage of PWID that initiate injecting aged 30–49 in 2018	20.7% (17.1–24.1%)	Normal	21.3 (18.1–23.6)	
Number of years in age group 15–24 years	7.2 years	N/A		UHS data (Kral et al., 2010). Note that PWID aged 15–24 years stay < 10 years as enter on average older than 15.
Number of years in age group 25–29	4.8 years	N/A		UHS data (Kral et al., 2010)
Number of years in age group 30–49	19.2 years	N/A		UHS data (Kral et al., 2010)
Years prior to 2017 when decrease in PWID initiation rate started	20–40	Uniform	31.2 (21.4–39.2)	Recruitment into injecting thought to have dropped in past, but uncertain, so large range assumed.
Number of PWID entering the model				Wide uninformative priors assumed. Used to calibrate the model to PWID population sizes. Note that number entering before decrease must be greater than the number entering after the decrease in the PWID initiation rate which is included in the ABC SMC procedure.
Before decrease in PWID initiation rate	100–3000	Uniform	2067 (989–2907)	
After decrease in PWID initiation rate	100–1500	Uniform	701 (460–906)	
Overall drug and nondrug related mortality rate per year,%	0.91 (0.59–1.25)	Normal	0.93 (0.65–1.23)	(Evans et al., 2012)
Temporary cessation rate per year for those aged 15–29 years	0.16 (0.1–0.2)	Uniform	0.16 (0.14–0.19)	(Evans et al., 2009)
Temporary cessation rate for those aged				Uninformative prior. Encompasses range of cessation rate of 15–29 yr olds.
30–49	0–0.5	Uniform	0.1 (0.01–0.2)	
50+	0–0.5	Uniform	0.3 (0.1–0.5)	
Permanent cessation rate for all PWID	0–0.4	Uniform	0.2 (0.1–0.3)	Uninformative prior
Relapse rate to injecting per year for those aged				(Evans et al., 2009)
15–29 years	0.6 (0.4–0.7)	Uniform	0.6 (0.4–0.7)	
≥30 years	0.3 (0.2–0.6)	Uniform	0.4 (0.2–0.5)	
Assortative mixing by age	0–0.5	Uniform	0.2 (0.02–0.4)	Uninformative prior. Used to calibrate to mixing data by age.
Transmission rate among PWID aged				Uninformative priors calibrated to prevalence data
< 30	0–0.5	Uniform	0.1 (0.2–0.4)	
≥30	0–0.5	Uniform	0.1 (0.03–0.2)	
*Housing parameters*				
Average duration of being unstably housed	1.3–16.3 years	Uniform	1.7 (1.3–3.6)	(Morris et al., 2020) Average duration in paper ranges from 6.3years (rate of movement 0.159) to 8.1years (rate of movement 0.1229). Halved lower rate for bound and multiplied higher bound rate by 5 to get a wider range. Posterior shows rate needs to be lower to achieve the high proportion unstably housed that is seen in San Francisco.
Rate of becoming unstably housed per year				
Pre-2010	0–1.5	Uniform	1.3 (1.0–2.8)	
Post-2010	0–2.0	Uniform	0.7 (0.6–1.6)	Uninformative prior
Proportion entering the population as unstably housed				Calibrated to achieve unstable housing dynamics 17/22 PWID who initiated injecting in the past year were unstably housed (UFO analysis, 2017 wave). Percentage who initiate injecting unstably housed thought to have increased in line with the proportion unstably housed.
Pre-2010	0.5–0.6	Uniform	0.5 (0.5–0.6)	
Post-2010	0.7–0.9	Uniform	0.8 (0.7–0.9)	Calibrated to achieve unstable housing dynamics.
Assortative mixing by unstable housing status	0–0.5	Uniform	0.2 (0.03–0.5)	Uninformative prior
Relative risk of acquiring HCV while unstably housed	1.7 (1.2–2.3)	Log-normal	1.5 (1.2–2.1)	(Morris et al., 2020)
Unadjusted RR associated with accessing testing if unstably housed	0.81 (0.72–0.92)	Log-normal	0.8 (0.7–0.9)	NHBS analysis
Unadjusted RR associated with accessing treatment if unstably housed	0.66 (0.52–0.84)	Log-normal	0.7 (0.5–0.8)	NHBS analysis
*MOUD parameters*				
Year MOUD started in San Francisco	2000	Point estimate		Coverage low before 2000 (UFO data)
Rate leave MOUD per year	1.0 (0.6–1.4)	Normal	1.0 (0.6–1.3)	(Bao et al., 2009) Gives 1 yr (7.5–18 months) on MOUD in USA studies in review
Relative risk of acquiring HCV while on MOUD	0.5 (0.4–0.63)	Log-normal	0.5 (0.4–0.6)	(Platt et al., 2018)
Rate PWID aged 18–29 initiate MOUD per year				
Pre-2004	0–0.5	Uniform	0.1 (0.02–0.1)	Uninformative prior. Calibrated to achieve MOUD coverage among YPWID.
Post-2004	0–0.5	Uniform	0.1 (0.07–0.2)	
Rate PWID aged 30–49 initiate MOUD per year Post-2004	0–0.5	Uniform	0.2 (0.1–0.4)	Note that the rate for PWID aged 30–49 and 50+ Pre-2004 is calculated so that the change in rate seen among YPWID is reflected among PWID aged ≥30.
Rate PWID aged 50+ initiate MOUD per year				
Post-2004	0–0.5	Uniform	0.2 (0.1–0.4)	
Hazard ratio for increase in temporary cessation if accessing MOUD	1.7 (1.4–2.1)	Uniform	1.8 (1.5–2.1)	(Xia et al., 2015)
Hazard ratio for increase in recruitment onto MOUD if previously accessed MOUD	1–5	Uniform	2.2 (1.1–3.9)	Uninformative prior
*HCV treatment and clearance parameters*				
SVR rate	85–95%	Uniform	90.6 (86.3–94.4)	(AASLD, 2017; Accessed on 30 August 2017.)
Duration of treatment, weeks	12	Point estimate		(AASLD, 2017; Accessed on 30 August 2017.)
Treatment year start	2016	Point estimate		
Average proportion of infections that clear spontaneously	0.2–0.5	Uniform	0.3 (0.2–0.5)	Widened range from Micallef (Micallef et al., 2006).
Average proportion of re-infections that clear spontaneously (if have not been previously treated)	0.62–1	Uniform	0.8 (0.7–1.0)	(Vickerman et al., 2012)
Year testing and treatment started	2014–2016	Uniform	2015 (2014–2016)	
Testing rate among those aged				Uninformative priors calibrated to the cascade of care which differs by age.
<30	0–1	Uniform	0.6 (0.3–0.9)	
30–49	0–1	Uniform	0.7 (0.3–0.9)	
≥50	0–1	Uniform	0.6 (0.3–0.9)	
Treatment rate among those aged				Uninformative priors calibrated to the cascade of care which differs by age.
<30	0–1	Uniform	0.6 (0.2–0.9)	
30–49	0–1	Uniform	0.6 (0.3–1.0)	
≥50	0–1	Uniform	0.7 (0.3–1.0)	

**Table 2:** Model calibration data with uncertainty bounds.

Parameter	n/N	Range	Notes/References
HCV care cascade/prevalence and incidence data			
HCV antibody prevalence			
among those aged 30–49 in 2018	159/220	72.3% (65.9–78.1%)	NHBS analysis
among those aged ≥50 in 2018	154/191	80.6% (74.3–86.0%)	NHBS analysis
HCV RNA confirmed and aware of status among those with confirmed infections (proportion diagnosed)			NHBS analysis
aged 15–29 in 2018	18/25	72.0% (50.6–87.9%)	
aged 30–49 in 2018	93/121	76.9% (68.3–84.0%)	
aged ≥50 in 2018	86/114	75.4% (66.5–83.0%)	
HCV treated among those aware of their status			
aged 15–29 in 2018	6/18	33.3% (13.3–59.0%)	
aged 30–49 in 2018	48/93	51.6% (41.0–62.1%)	NHBS analysis
aged ≥50 in 2018	52/86	60.4% (49.3–70.8%)	
HCV incidence among YPWID in			
2001		25.1/100pyrs (18.7–32.9)	(Hahn et al., 2002)
Mid-2006		23.1/100pyrs (19.9–26.9)	(Page et al., 2013)
Population sizes			
Population size of YPWID in 2017.		2516–4979	(Facente et al., 2021) Estimate for 2015–2019 so have taken the mid-point.
Total PWID population size in 2015		14,037–39,946	(Facente et al., 2018) Calibrated to 2015 as estimate used two estimated specific to this year in their analysis.
Percentage of PWID aged *>*30 who are aged 30–49 in 2018. MOUD coverage		48.6–58.4%	NHBS 2018 data analysed for this project.
Percentage of YPWID accessing MOUD in the past 3 months			
2004	34/1294	2.6% (1.8–3.6%)	2004 and 2015 estimates from UFO analysis (Fraser et al., 2019)
2015	125/1025	12.2% (10.3–14.4%)	2017 estimate from UFO analysis for this project
2017	35/295	11.9% (8.4–16.1%)	
Percentage of PWID of a given age accessing MOUD in the past 12 months in 2018			
YPWID	13/51	25.5% (14.3–39.6%)	NHBS 2018 data analysed for this project.
30–49 yr olds	100/217	46.1% (39.3–53.0%)	
50+yr olds	83/187	44.4% (37.1–51.8%)	
Percentage of YPWID ever having accessed MOUD in 2017 Unstable housing	94/295	31.9% (26.6–37.5%)	UFO analysis (2017 wave) for this project.
Percentage of PWID unstably housed in			(Coffin et al., 2015)
2005	319/565	56.5% (52.3–60.6%)	2017/18–UFO and NHBS analysis.
2009	299/535	55.9% (51.6–60.1%)	Note that estimates for 2005/2009/2012 are currently homeless rather than unstable housing, but 2017/18 data show
2012	345/570	60.5% (56.4–64.6%)	similar percentages for both so have used as a proxy for model calibration.
2017	222/295	75.3% (70.0–80.1%)	2017 data is for YPWID only, but similar trend in unstable housing among YPWID and all PWID so have included.
2018	335/454	73.8% (69.5–77.8%)	
Mixing parameters			
Percentage of mixing being like-with-like by age among YPWID	N/A	54–62%	Analysis from UFO data (Fraser et al., 2019)

**Table 3:** Model projected outcomes for all PWID, YPWID and unstably housed PWID for each modelled scenario for: Incidence per 100 person years in 2030; percentage decrease in incidence over 2015–2030; probability of achieving the elimination goal of an 80% reduction in HCV incidence over 2015–2030; and median year when the elimination goal of an 80% reduction in HCV incidence since 2015 is achieved. Note that in 2015 the incidence is 9.6/100 pyrs (5.0–15.8) among all PWID, 23.5 (18.2–30.6) among YPWID and 10.8 (5.6–17.9) among unstably housed PWID.

	Incidence in 2030 (/100pyrs)	% decrease in incidence over 2015–2030	Number of treatments over 2015–2030	Number of treatments over 2025–2030	Probability of achieving elimination goal by 2030	Median (95%CrI) year elimination goal of an 80% reduction in HCV incidence since 2015 is achieved
*All PWID*						
*Status quo*	1.5 (0.3–4.4)	83.3 (60.6–96.9)	12,421 (7,017–23,920)	1,618 (795–3,156)	62.0%	2028.5 (2022.5–2051.0)
*Scenario 1*	1.0 (0.3–2.5)	89.5 (78.5–97.2)	12,964 (7,645–24,368)	1,553 (815–3,054)	95.7%	2025.5 (2022.0–2031.0)
*Scenario 2*	1.4 (0.2–4.1)	84.8 (62.0–97.5)	12,546 (7,101–24,085)	1,743 (854–3,388)	67.5%	2028.0 (2022.5–2051.0)
*Scenario 3*	1.3 (0.2–3.8)	86.5 (66.0–97.5)	12,400 (7,003–23,907)	1,599 (784–3,132)	75.9%	2027.0 (2022.5–2045.0)
*Scenario 4*	1.1 (0.2–3.6)	87.7 (67.6–98.0)	12,524 (7,079–24,064)	1,721 (837–3,364)	80.0%	2027.0 (2022.5–2040.0)
*Scenario 5*	1.2 (0.2–3.3)	86.9 (70.7–97.6)	12,695 (7,301–24,218)	1,852 (830–3,773)	80.9%	2027.0 (2022.5–2036.0)
*Scenario 6*	0.9 (0.1–2.7)	89.8 (75.9–98.3)	12,911 (7,559–24,380)	1,972 (833–3,973)	92.4%	2026.5 (2022.5–2032.0)
*Scenario 7*	1.1 (0.2–3.0)	88.4 (72.5–98.1)	12,839 (7,405–24,377)	1,994 (887–3,985)	86.0%	2027.0 (2022.5–2034.0)
*Scenario 8*	0.8 (0.1–2.4)	91.5 (78.1–98.8)	13,048 (7,684–24,598)	2,108 (876–4,220)	95.6%	2026.5 (2022.5–2031.0)
*YPWID*						
*Status quo*	4.4 (0.9–10.7)	81.3 (57.0–96.2)	1,551 (574–2,825)	287 (136–536)	54.8%	2029.5 (2023.0–2051.0)
*Scenario 1*	2.7 (0.8–6.2)	88.3 (76.0–96.6)	1,692 (684–2,943)	303 (161–539)	91.3%	2026.5 (2023.0–2032.5)
*Scenario 2*	4.0 (0.7–10.4)	83.0 (58.7–97.0)	1,594 (595–2,888)	326 (150–606)	60.9%	2029.0 (2023.0–2051.0)
*Scenario 3*	3.3 (0.6–8.8)	85.8 (64.9–97.1)	1,538 (571–2,803)	274 (132–514)	72.9%	2027.5 (2023.0–2044.0)
*Scenario 4*	3.0 (0.5–8.4)	87.2 (66.3–97.8)	1,578 (592–2,862)	310 (146–578)	77.6%	2027.0 (2023.0–2039.5)
*Scenario 5*	3.5 (0.7–8.1)	85.3 (67.8–97.0)	1,614 (640–2,880)	341 (174–617)	73.3%	2028.0 (2023.0–2037.0)
*Scenario 6*	2.5 (0.4–6.3)	89.2 (75.1–98.1)	1,861 (863–3,189)	481 (217–895)	90.5%	2027.0 (2023.0–2032.0)
*Scenario 7*	3.1 (0.5–7.6)	87.1 (69.9–97.7)	1,661 (662–2,943)	385 (190–694)	79.7%	2027.5 (2023.0–2035.0)
*Scenario 8*	2.1 (0.3–5.6)	91.0 (77.8–98.7)	1,920 (896–3,270)	534 (233–991)	94.6%	2026.5 (2023.0–2031.0)
*Unstably housed PWID*						
*Status quo*	1.6 (0.3–4.6)	84.5 (63.5–97.1)	6,696 (2,691–13,810)	1,119 (559–2,195)	67.6%	2027.5 (2022.5–2051.0)
*Scenario 1*	1.1 (0.3–2.9)	89.4 (78.3–97.1)	5,732 (2,197–11,911)	684 (341–1,392)	95.2%	2025.5 (2022.0–2031.5)
*Scenario 2*	1.5 (0.2–4.4)	85.9 (65.0–97.6)	6,790 (2,743–13,934)	1,205 (600–2,337)	72.8%	2027.0 (2022.5–2050.0)
*Scenario 3*	1.3 (0.2–3.9)	87.4 (69.1–97.7)	6,683 (2,685–13,795)	1,105 (551–2,177)	80.5%	2026.5 (2022.5–2042.0)
*Scenario 4*	1.2 (0.2–3.8)	88.6 (70.4–98.2)	6,774 (2,736–13,915)	1,188 (592–2,317)	84.0%	2026.5 (2022.5–2038.0)
*Scenario 5*	1.4 (0.2–3.8)	86.8 (70.3–97.5)	6,433 (2,508–13,395)	860 (426–1,730)	80.1%	2027.0 (2022.5–2036.0)
*Scenario 6*	1.1 (0.2–3.1)	89.7 (75.7–98.3)	6,722 (2,983–13,533)	1,040 (518–1,966)	92.2%	2026.5 (2022.5–2032.0)
*Scenario 7*	1.2 (0.2–3.5)	88.3 (72.1–98.1)	6,506 (2,545–13,497)	924 (453–1,846)	85.3%	2027.0 (2022.5–2034.0)
*Scenario 8*	0.9 (0.1–2.8)	91.4 (77.8–98.8)	6,804 (3,041–13,642)	1,118 (544–2,108)	95.3%	2026.5 (2022.5–2031.0)

Scenarios are: Status quo: Continuing with testing and treatment as during COVID; Scenario 1: Counterfactual of no change in testing and treatment due to the pandemic from March 2020; Scenario 2: Testing and treatment levels return to pre-pandemic levels by 2025 among all PWID (linear increase over 2022–2025 from 59.1% decrease seen during pandemic); Scenario 3: MOUD levels increase over 2024–2025 among YPWID (from 25.5% (14.3–39.6%) accessing MOUD in last year to 46.1% (39.3–53.0%); same as among PWID aged 30–49) and sustained thereafter; Scenario 4: Scenario 2 plus Scenario 3; Scenario 5: Unstable housing levels decrease linearly over 2024–2025 among all PWID (from 87.6% to pre-pandemic level of 73.8% [95% CrI: 69.5–77.8%])) Scenario 6: Scenario 5 and increase in HCV testing and treatment levels in 2024 among unstably housed PWID to same as stably housed PWID; Scenario 7: Scenario 2 plus Scenario 5; and Scenario 8: Scenario 2 plus Scenario 6.
