# Supplementary material for: Modeling the impact of the COVID-19 pandemic on achieving HCV elimination amongst young and unstably housed people who inject drugs in San Francisco
Source: Int J Drug Policy. Author manuscript; Available in PMC 2024 Dec 6. (PMC7617003; doi:10.1016/j.drugpo.2024.104452)
Supplement: Supplementary file [file EMS200025-supplement-Supplementary_file.docx]

# Supplementary Materials for Modeling the impact of the COVID-19 pandemic on achieving HCV elimination amongst young and unstably housed people who inject drugs in San Francisco

Hannah Fraser^1^, Jack Stone^1^, Shelley N Facente^2,3^, Adelina Artenie^1^, Sheena Patel^4^, Erin C Wilson^5^, Willi McFarland^4,5^, Kimberly Page^6^, Peter Vickerman^1^*, Meghan D Morris^4^*

^1^ Population Health Sciences, Bristol Medical School, University of Bristol, UK

^2^ School of Public Health, Division of Epidemiology and Biostatistics, University of California Berkeley, Berkeley, USA

^3^ Facente Consulting, Richmond, USA

^4^ Department of Epidemiology and Biostatistics, University of California San Francisco, San Francisco, USA

^5^ San Francisco Department of Public Health, San Francisco, USA

^6^ Department of Internal Medicine, Division of Epidemiology, University of New Mexico, USA.

* Co-senior author

## Supplementary Tables

**Supplementary Table 1**: Details of the different studies that were used to parameterise the models.

| Study | Years | Sample size at enrolment | Brief study details | Reference(s) |
| --- | --- | --- | --- | --- |
| UFO Study | 1997 - 2018 | 295 for current analysis | Longitudinal study.  Young PWID (aged < 30) recruited by outreach worked. Inclusion criteria included being aged < 30, self-reported injecting the past 30 days, and post-2005 whether self-reported HCV-negative or unknown status. | (Hahn et al., 2002; Morris et al., 2020; Page et al., 2009) |
| National HIV Behavioural Surveillance (NHBS) System for PWID | 2005; 2009; 2012; 2015; 2018. | 565 (2005); 535 (2009); 570 (2012); 538 (2015); 464 (2018) | Cross sectional survey used to monitor HIV prevalence and risk in high-risk groups. Recruitment via respondent driven sampling. Inclusion criteria included being aged $\geq$18, injecting illicit substances in the past 12 months and having a valid study coupon. | (CDC, 2015, 2018 2020; Coffin et al., 2015; Kral et al., 2010) |
| Urban Health Study | 1986 - 2005 | Data for 2,296 PWID between 1998-2000 | Targeted sampling methods from 6 different inner-city San Francisco Bay area neighbourhoods. Inclusion criteria included being aged $\geq$18, injecting illicit drugs within the past 30 days or previously being enrolled in UHS. | (Tseng et al., 2007) |
| No One Waits Study (NOW) | 2020-2021 | Data for 492 PWID in June 2021 | Street-outreach recruitment targeted people experiencing homelessness and injecting drugs for rapid HCV antibody (anti-HCV) testing followed by confirmatory HCV RNA testing if positive. | (Morris et al., 2023) |

**Supplementary Figure 1**: A comparison of the baseline calibrated model (not including effect of COVID-19 on decreasing testing and treatment rates) with available data used to calibrate the model (given in Table 2 and shown as black points and whiskers). Black lines indicate the median model projections, while the pink shading is the 95% credibility intervals and the dashed lines are the Inter-quartile range.


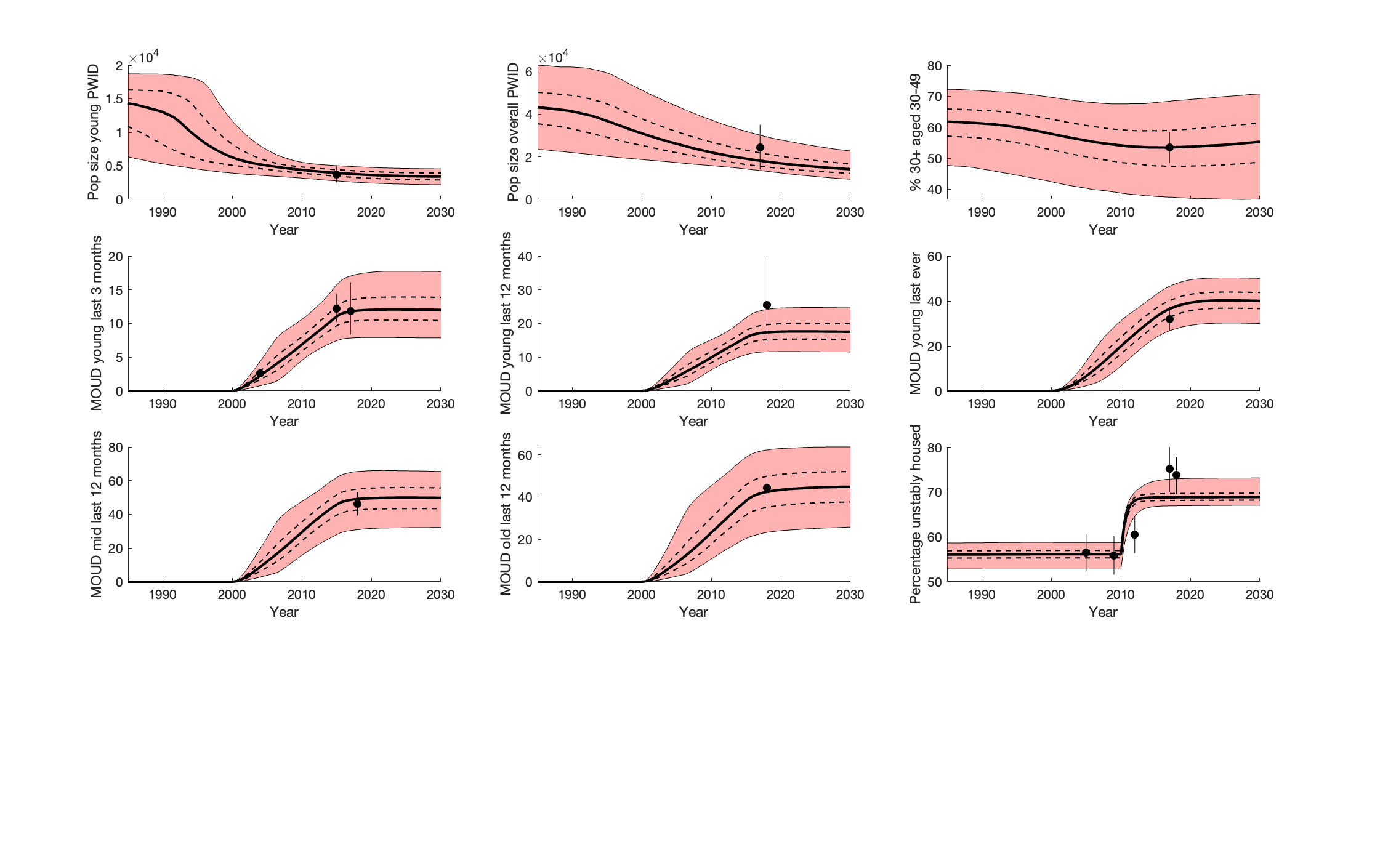


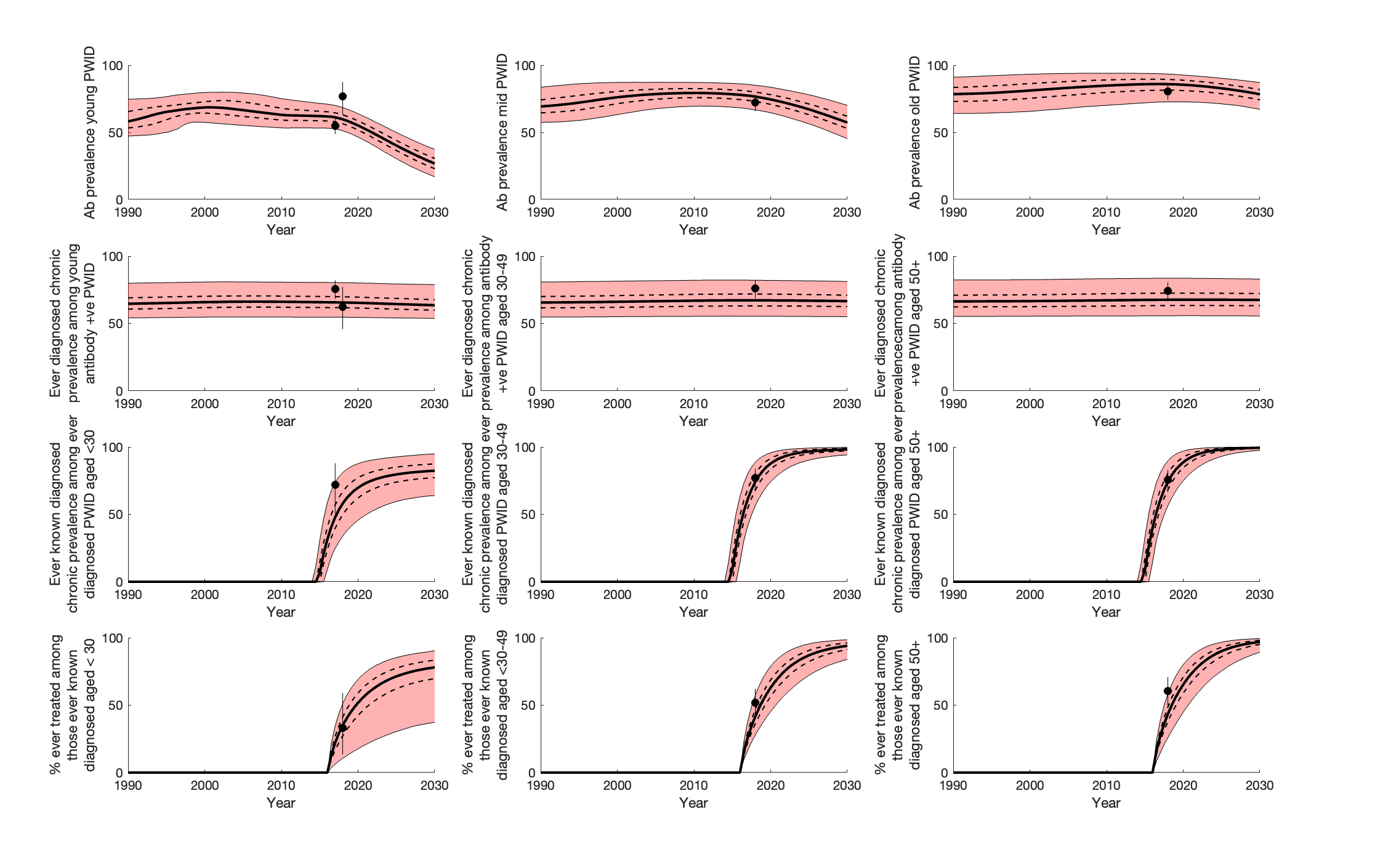


## Methods

### Model description

Additional assumptions not discussed in the main paper are given below:

- Due to the long timeframe associated with HCV disease progression and related mortality(Smith et al., 2015; Stone et al., 2021), we assumed no additional mortality due to HCV complications among people in the model, i.e. currently injecting or temporarily ceased.
- As syringe service programs (SSP) initiated in 1989(Hahn et al., 2001) and have had high and stable coverage since 1997(CDC, 2015, 2018 ; Hahn et al., 2001) we do not explicitly model this.
- We assume a linear increase/decrease in the proportion of PWID entering the model in the different age groups between 2010 and 2018 based on the sampled proportion in each.
- Additionally to the MOUD assumptions in the main paper, we assumed that 31.7% of YPWID had ever accessed MOUD in 2017
- We assume an immediate change in the proportion of PWID entering the model as unstably housed in 2010.

## Model equations

Let $X_{i,j,k}^{m,n}$ be the number of PWID in the model where

- subscript $i$ denotes age ($i=1$, aged 18-24; $i=2$, aged 25-29; $i=3$, aged 30-49; $i=4$, aged $\geq$50)
- subscript $j$ denotes injecting status ($j=1$, currently injecting; $j=2$, temporarily ceased injecting)
- subscript $k$ denotes MOUD status ($k=1$, never accessed MOUD; $k=2$, currently accessing MOUD; $k=3$, not on MOUD, accessed within the last 3 months; $k=4$, not on MOUD, accessed within the last 3-12 months; $k=5$, not on MOUD, accessed >12 months ago)
- superscript $m$ denotes housing status ($m=1$, stably housed; $m=2$, unstably housed)
- superscript $n$ denotes HCV infection stage ($n=1$, susceptible to HCV (Ab- RNA-); $n=2$, susceptible to HCV after spontaneous clearance (Ab+ RNA-); $n=3$, HCV chronic undiagnosed infection (Ab+ RNA+); $n=4$, HCV chronic diagnose infection (Ab+ RNA+); $n=5$, accessing HCV treatment (Ab+ RNA-); $n=6$, HCV chronic infection having failed treatment (Ab+ RNA+); $n=7$, susceptible to HCV having previously been cured (Ab+ RNA-); $n=8$, HCV chronic undiagnosed infection (reinfection, Ab+ RNA+); $n=9$, HCV chronic diagnosed infection (reinfection, Ab+ RNA+).

The ordinary differential equation model can be written as:

$$\frac{dX_{i,j,k}^{m,n}}{dt}=\Theta_{i,j,k}^{m,n}+M_{i,j,k}^{m,n}+A_{i,j,k}^{m,n}+T_{i,j,k}^{m,n}+\Sigma_{i,j,k}^{m,n}+H_{i,j,k}^{m,n}+\Pi_{i,j,k}^{m,n}+\Lambda_{i,j,k}^{m,n}$$

where

- $\Theta_{i,j,k}^{m,n}$ represents recruitment into the model
- $M_{i,j,k}^{m,n}$ represents background mortality and permanent cessation
- $A_{i,j,k}^{m,n}$ represents aging in the model
- $T_{i,j,k}^{m,n}$ represents transitions between current and temporarily ceased injectors (temporary cessation and relapse)
- $\Sigma_{i,j,k}^{m,n}$ represents transitions between MOUD states
- $H_{i,j,k}^{m,n}$ represents transitions between housing states
- $\Lambda_{i,j,k}^{m,n}$ represents HCV transmission
- $\Pi_{i,j,k}^{m,n}$ represents HCV transitions (due to screening and treatment)

### Recruitment into the model

The inflow of new injectors is given by

$\Theta_{i,1,1}^{1,1}=\left( 1-p\left( t \right) \right)q_{i}\left( t \right)\theta\left( t \right)$ for $i=1,2,3$

$\Theta_{i,1,1}^{2,1}=p{\left( t \right)q}_{i}\left( t \right)\theta\left( t \right)$ for $i=1,2,3$

$\Theta_{i,j,k}^{m,n}=0$ for all other combinations of $i,j,k,m,n$

where:

- $p$ is the proportion of newly injecting PWID who enter the model unstably housed at time $t$
- $q_{i}(t)$ is the proportion of newly injecting PWID who enter the model into age group $i$ at time $t$
- $\theta(t)$ is the number of new PWID who enter the model at time $t$

### Background mortality and permanent cessation

Background mortality and permanent cessation of injecting is given by

$$M_{i,1,k}^{m,n}=-\mu X_{i,1,k}^{m,n}$$

$$M_{i,2,k}^{m,n}=-\left( \mu+\bar{\mu} \right)X_{i,2,k}^{m,n}$$

where:

- $\mu$ is the background mortality rate
- $\bar{\mu}$ is the permanent cessation rate from injecting

### Aging

Aging between the different age groups is given by

$$A_{1,j,k}^{m,n}=-\frac{1}{a_{1}}X_{1,j,k}^{m,n}$$

$A_{i,j,k}^{m,n}=\frac{1}{a_{i-1}}X_{i,j,k}^{m,n}-\frac{1}{a_{i}}X_{i,j,k}^{m,n}$ for $i=2,3$

$$A_{4,j,k}^{m,n}=\frac{1}{a_{3}}X_{3,j,k}^{m,n}$$

where:

- $a_{i}$ is the average duration of staying in age group $i$

### Transitions between current and temporarily ceased injectors

Relapse and temporary cessation is given by

$$T_{i,1,k}^{m,n}=-\phi_{i}X_{i,1,k}^{m,n}+\rho_{i}X_{i,2,k}^{m,n}$$

$$T_{i,2,k}^{m,n}=\phi_{i}X_{i,1,k}^{m,n}-\rho_{i}X_{i,2,k}^{m,n}$$

where:

- $\phi_{i}$ is the temporary cessation rate of PWID in age group $i$. Note that $\phi_{1}=\phi_{2}$.
- $\rho_{i}$ is the relapse rate of PWID in age group $i$. Note that $\rho_{1}=\rho_{2}$ and $\rho_{3}=\rho_{4}$.

### Transitions between MOUD states

Transitions between MOUD states are given by

$$\Sigma_{i,j,1}^{m,n}=-\beta_{i}X_{i,j,1}^{m,n}$$

$$\Sigma_{i,j,2}^{m,n}=\beta_{i}X_{i,j,1}^{m,n}+\hat{r}\beta_{i}\sum_{l=3,4,5} X_{i,j,l}^{m,n}-\gamma X_{i,j,2}^{m,n}$$

$$\Sigma_{i,j,3}^{m,n}=\gamma X_{i,j,2}^{m,n}-\left( {\hat{r}\beta}_{i}+\frac{1}{0.25} \right)X_{i,j,3}^{m,n}$$

$$\Sigma_{i,j,4}^{m,n}=\frac{1}{0.25}X_{i,j,3}^{m,n}-\left( {\hat{r}\beta}_{i}+\frac{1}{0.75} \right)X_{i,j,4}^{m,n}$$

$$\Sigma_{i,j,5}^{m,n}=\frac{1}{0.75}X_{i,j,4}^{m,n}-{\hat{r}\beta}_{i}X_{i,j,5}^{m,n}$$

where:

- $\beta_{i}$ is the rate of recruitment onto MOUD for age group $i$. Note that $\beta_{1}=\beta_{2}$.
- $\hat{r}$ is the increase in recruitment rate onto MOUD if PWID have previously ever been on MOUD.
- $\gamma$ is the MOUD leaving rate
- Note that 1/0.25 and 1/0.75 represent the average duration of being in these compartments (3 months and 9 months, respectively).

Note that

- We assume that there is a different rate of recruitment onto MOUD for ages 18-29 years, 30-49 years and 50+ years.
- We assume a linear increase in the rate of recruitment onto MOUD between 2000-2006 and a second linear increase between 2006-2017, after which the recruitment rate is stable.
- Due to the data that we have we estimate both of these rates for those aged 18-29 years. For those aged $\geq$30 years we estimate the rate of recruitment in 2004 by assuming that the ratio of rates of those aged <30 between 2017 and 2014 is the same as the ratio of rates in those aged $\geq$30 between 2017 and 2014. i.e. over time for those aged < 30 the rate of recruitment is given by the following, where $b_{1}$ and $b_{2}$ are calibrated such that the coverages of MOUD seen in the data are achieved.

$$\beta_{1}=\left\{ \begin{matrix} 0 & if t<2000 \\ \frac{\left( t-2000 \right)b_{a}^{1}}{4} & it 2000\leq t<2004 \\ b_{a}^{1}+\frac{\left( t-2004 \right)b_{b}^{1}}{11} & if 2004\leq t<2015 \\ b_{b}^{1} & if t\geq2015 \end{matrix} \right.$$

Note that the above also holds for $\beta_{2}$ as $\beta_{1}=\beta_{2}$. For $\beta_{3}$ we have that $b_{b}^{3}$ is sampled and calibrated to the data, and

$$b_{a}^{3}=\frac{b_{b}^{3}}{S}$$

where $S$ is the scaling factor $\frac{b_{b}^{1}}{b_{a}^{1}}$. This is determined similarly for $b_{a}^{4}$.

### Transition between housing states

Transitions between stable and unstable housing states (within the model) are given by

$$H_{i,j,k}^{1,n}=h_{r}X_{i,j,k}^{2,n}-h_{l}X_{i,j,k}^{1,n}$$

$$H_{i,j,k}^{2,n}=-h_{r}X_{i,j,k}^{2,n}+h_{l}X_{i,j,k}^{1,n}$$

where:

- ${1/h}_{r}$ is the average duration that PWID are unstably housed
- $1/h_{l}$ is the average duration that PWID are stably housed

### HCV transmission

HCV infection is given by

$$\Lambda_{i,1,k}^{m,1}=-\lambda_{w,k,m}X_{i,1,k}^{m,1}$$

$$\Lambda_{i,2,k}^{m,2}=\delta_{1}\lambda_{w,k,m}X_{i,1,k}^{m,1}-\left( 1-\delta_{2} \right)\lambda_{w,k,m}X_{i,1,k}^{m,2}$$

$$\Lambda_{i,1,k}^{m,3}=\left( 1-\delta_{1} \right)\lambda_{w,k,m}X_{i,1,k}^{m,1}+\left( 1-\delta_{2} \right)\lambda_{w,k,m}X_{i,1,k}^{m,2}$$

$$\Lambda_{i,1,k}^{m,7}=-\left( 1-\delta_{1} \right)\lambda_{w,k,m}X_{i,1,k}^{m,7}$$

$$\Lambda_{i,1,k}^{m,8}=\left( 1-\delta_{1} \right)\lambda_{w,k,m}X_{i,1,k}^{m,7}$$

$$\Lambda_{i,2,k}^{m,n}=0$$

where:

- $\lambda_{w,k,m}$ is the HCV force of infection for PWID in age group $w$, MOUD state $k$ and housing state $m$. Note that age groups have been separated into $<30$($w=0 if i=1,2$) and $\geq30$ ($w=1 if i=3,4$)
- $\delta_{1}$ is the proportion of HCV infections that spontaneously clear if a primary infection or previously treated and $\delta_{2}$ is the proportion of HCV infections that spontaneously clear if have previously spontaneously cleared primary infection.

#### HCV force of infection

We assume like-with-like mixing among different ages and those in different housing states. The model assumes two transmission rates; one among young adult PWID aged < 30 years, and a second among PWID aged $\geq30$ years. For those not accessing MOUD the force of infection is given by:

$$\lambda_{w,1,1}=\pi_{w}(v_{r}\left( v_{a}H_{w,1}+\left( 1-v_{a} \right)H_{*,1} \right)+\left( 1-v_{r} \right)\left( v_{a}H_{w,*}+\left( 1-v_{a} \right)H_{*,*} \right))$$

$$\lambda_{w,1,2}=\pi_{w}\psi(v_{r}\left( v_{a}H_{w,2}+\left( 1-v_{a} \right)H_{*,2} \right)+\left( 1-v_{r} \right)\left( v_{a}H_{w,*}+\left( 1-v_{a} \right)H_{*,*} \right))$$

where:

- $\pi_{w}$ is the transmission rate among those in age group $w$
- $v_{r}$ is the like-with-like mixing among those stably/unstably housed
- $v_{a}$ is the like-with-like mixing among YPWID and PWID aged $\geq$30 years
- $H$ represents the mixing between the different age groups $w$ and housing states $m$ which is explained further below
- $\psi$ is the relative increase in HCV transmission is unstably housed.
- Note
  - $\Gamma$ (used below) is the relative reduction in HCV transmission if accessing MOUD.
  - $\alpha$ (used below) is the sustained virological response rate (SVR) among those undertaking HCV treatment.

Let $Y_{w,k}^{m,r}$ represent the number of current PWID in age group $w$ (previously defined), MOUD state $k$ (previously defined), housing state $m$ (previously defined) and infection state $r$ ($r=1$ chronically infected with HCV; $i=2$ susceptible to HCV (with or without antibodies) such that

$$Y_{w,k}^{m,1}=\sum_{l=3,4,6,8,9} Z_{w,1,k}^{m,l}+\left( 1-\alpha\right)Z_{w,1,k}^{m,5}$$

$$Y_{w,k}^{m,2}=\sum_{l=1,2,7} Z_{w,1,k}^{m,n}+\alpha Z_{w,1,k}^{m,5}$$

where

$Z_{1,1,k}^{m,n}=\sum_{l=1,2} X_{l,1,k}^{m,n}$ and $Z_{2,1,k}^{m,n}=\sum_{l=3,4} X_{l,1,k}^{m,n}$

We then have the following assortative and random mixing terms which can be substituted into the equations above.

1. Assortative mixing by housing status is represented by:

$$H_{*,h}=\frac{\sum_{w} \left( \sum_{l=1,3:5} Y_{w,l}^{h,1}+\Gamma Y_{w,2}^{h,1} \right)}{\sum_{s} \sum_{w} (\sum_{l=1,3:5} Y_{w,l}^{h,s}+\Gamma Y_{w,2}^{h,s})}$$

2. Assortative mixing by age is represented by:

$$H_{h,*}=\frac{\sum_{l=1,3:5} Y_{h,l}^{1,1}+\psi\sum_{l=1,3:5} Y_{h,l}^{2,1}+\Gamma(Y_{h,2}^{1,1}+\psi Y_{h,2}^{2,1})}{\sum_{s} \left( \sum_{l=1,3:5} Y_{h,l}^{1,s}+\psi\sum_{l=1,3:5} Y_{h,l}^{2,s}+\Gamma(Y_{h,2}^{1,s}+\psi Y_{h,2}^{2,s}) \right)}$$

3. Assortative mixing by age and housing status is represented by:

$$H_{w,m}=\frac{\sum_{l=1,3:5} Y_{w,l}^{m,1}+\Gamma Y_{w,2}^{m,1}}{\sum_{s} \left( \sum_{l=1,3:5} Y_{w,l}^{m,s}+\Gamma Y_{w,2}^{m,s} \right)}$$

4. Fully random mixing is represented by

$$H_{*,*}=\frac{\sum_{w} \sum_{l=1,3:5} Y_{w,l}^{1,1}+\psi\sum_{w} \sum_{l=1,3:5} Y_{w,l}^{2,1}+\Gamma\left( \sum_{w} Y_{w,2}^{1,1}+\psi\sum_{w} Y_{w,2}^{2,1} \right)}{\sum_{s} \left( \sum_{w} \sum_{l=1,3:5} Y_{w,l}^{1,s}+\psi\sum_{w} \sum_{l=1,3:5} Y_{w,l}^{2,s}+\Gamma\left( \sum_{w} Y_{w,2}^{1,s}+\psi\sum_{w} Y_{w,2}^{2,s} \right) \right)}$$

As we assume that the risk parameters act on both susceptibility and infectivity we finally have that

$\lambda_{w,k,m}=\lambda_{w,k,m}$ if $k=1,3,4,5$

$\lambda_{w,k,m}=\Gamma\lambda_{w,k,m}$ if $k=2$

### HCV transitions (due to screening and treatment)

Transitions due to HCV screening and treatment are given by

$$\Pi_{i,j,k}^{m,3}=-\kappa_{i}^{m}Y_{i,j,k}^{m,3}$$

$$\Pi_{i,j,k}^{m,4}=\kappa_{i}^{m}Y_{i,j,k}^{m,3}-\eta_{i}^{m}Y_{i,j,k}^{m,4}$$

$$\Pi_{i,j,k}^{m,5}=\eta_{i}^{m}\sum_{l=4,6,9} Y_{i,j,k}^{m,l}-\omega Y_{i,j,k}^{m,5}$$

$$\Pi_{i,j,k}^{m,6}=\left( 1-\alpha\right)\omega Y_{i,j,k}^{m,5}-\eta_{i}^{m}Y_{i,j,k}^{m,6}$$

$$\Pi_{i,j,k}^{m,7}=\alpha\omega Y_{i,j,k}^{m,6}$$

$$\Pi_{i,j,k}^{m,8}=-\kappa_{i}^{m}Y_{i,j,k}^{m,8}$$

$$\Pi_{i,j,k}^{m,9}=\kappa_{i}^{m}Y_{i,j,k}^{m,8}-\eta_{i}^{m}Y_{i,j,k}^{m,9}$$

where:

- $\kappa_{i}^{m}$ is the time, age and housing status dependent HCV screening rate
- $\eta$ is the time, age and housing status dependent HCV treatment rate
- $\alpha$ is the proportion of PWID who achieve a sustained virological response (SVR) after treatment
- $\omega$ is the duration of treatment

All modelling was performed using MATLAB, version 2021a (Mathworks). The time horizon for model runs was 2050 with model outputs being produced every 1/20^th^ of year and a smaller adaptive time step being used to solve the model numerically.

## Model calibration

The model was calibrated using an approximate Bayesian computation Sequential Monte Carlo (ABC SMC) method to calculate summary statistics (Table 2, including data sources and parameter prior probability distributions) up to 2018 on: (1) the population size of YPWID in 2017; (2) the overall PWID population size in 2015; (3) the percentage of those aged $\geq$30 who are 30-49 years in 2017; (4) the proportion of mixing that occurs between YPWID; (5) the proportion of YPWID accessing MOUD in the past 3 months in 2004, 2014 and 2017; (6) the proportion of YPWID accessing MOUD in the past 12 months in 2018; (7) the proportion of YPWID who have ever accessed MOUD by 2017; (8) the proportion of PWID aged 30-49 accessing MOUD in the past 12 months in 2018; (9) the proportion of PWID aged 50+ accessing MOUD in the past 12 months in 2018; (10) HCV incidence among YPWID in 2001 and mid-2006; (11)-(12) anti-HCV prevalence among PWID aged 30-49 and PWID aged 50+ in 2018, respectively; (13)-(15) the proportion of YPWID, PWID aged 30-49 and PWID aged 50+ who have ever had chronic infection (diagnosed and undiagnosed), respectively in 2018; (16)-(18) the proportion of YPWID, PWID aged 30-49 and PWID aged 50+ who have been diagnosed in 2018, (19)-(21) the proportion of YPWID, PWID aged 30-49 and PWID aged 50+ who have ever been treated in 2018 among those diagnosed; and (22) the proportion of PWID unstably housed in 2005, 2009, 2012, 2017 and 2018.

We performed the ABC SMC by sampling 5,000 parameter sets, which are iteratively perturbed to improve the goodness of fit until we achieve our desired criteria (population sizes and mixing and incidence had to be within required 95% bounds; likelihoods for other estimates had to be >95% difference between the first iteration and the current iteration).

## References

CDC. (2015). *HIV Infection, Risk, Prevention, and Testing Behaviors among Persons Who Inject Drugs—National HIV Behavioral Surveillance: Injection Drug Use, 20 U.S. Cities, 2012*. Retrieved 09/03/2018 from

CDC. (2018 (Archived at <http://www.webcitation.org/75T3qD4if> on 16/01/2019)). *HIV Infection, Risk, Prevention, and Testing Behaviors among Persons Who Inject Drugs—National HIV Behavioral Surveillance: Injection Drug Use, 20 U.S. Cities, 2015.* .

CDC. (2020). *HIV Infection Risk, Prevention, and Testing Behaviors among Persons Who Inject Drugs—National HIV Behavioral Surveillance: Injection Drug Use, 23*

*U.S. Cities, 2018. HIV Surveillance Special Report 24.*

Coffin, P. O., Jin, H., Huriaux, E., Mirzazadeh, A., & Raymond, H. F. (2015). Trends in use of health care and HIV prevention services for persons who inject drugs in San Francisco: results from National HIV Behavioral Surveillance 2005-2012. *Drug Alcohol Depend*, *146*, 45-51. <https://doi.org/10.1016/j.drugalcdep.2014.10.025>

Hahn, J. A., Page-Shafer, K., Lum, P. J., Bourgois, P., Stein, E., Evans, J. L., Busch, M. P., Tobler, L. H., Phelps, B., & Moss, A. R. (2002). Hepatitis C virus seroconversion among young injection drug users: relationships and risks. *The Journal of infectious diseases*, *186*(11), 1558-1564.

Hahn, J. A., Page-Shafer, K., Lum, P. J., Ochoa, K., & Moss, A. R. (2001). Hepatitis C virus infection and needle exchange use among young injection drug users in San Francisco. *Hepatology*, *34*(1), 180-187. <https://doi.org/10.1053/jhep.2001.25759>

Kral, A. H., Malekinejad, M., Vaudrey, J., Martinez, A. N., Lorvick, J., McFarland, W., & Raymond, H. F. (2010). Comparing respondent-driven sampling and targeted sampling methods of recruiting injection drug users in San Francisco. *J Urban Health*, *87*(5), 839-850. <http://www.ncbi.nlm.nih.gov/pubmed/20582573>

Morris, M., McDonell, C., Luetkemeyer, A., Thawley, R., McKinney, J., & Price, J. (2023). Community-Based Point-of-Diagnosis Hepatitis C Treatment for Marginalized Populations - A Nonrandomized Controlled Trial. *JAMA Netw Open*, *6*(10), e2338792.

Morris, M. D., Yen, I. H., Shiboski, S., Evans, J. L., & Page, K. (2020). Housing Stability and Hepatitis C Infection for Young Adults Who Inject Drugs: Examining the Relationship of Consistent and Intermittent Housing Status on HCV Infection Risk. *Journal of Urban Health*, *97*(6), 831-844. <https://doi.org/10.1007/s11524-020-00445-7>

Page, K., Hahn, J. A., Evans, J., Shiboski, S., Lum, P., Delwart, E., Tobler, L., Andrews, W., Avanesyan, L., Cooper, S., & Busch, M. P. (2009). Acute hepatitis C virus infection in young adult injection drug users: a prospective study of incident infection, resolution, and reinfection. *J Infect Dis*, *200*(8), 1216-1226. <https://doi.org/10.1086/605947>

Smith, D. J., Combellick, J., Jordan, A. E., & Hagan, H. (2015). Hepatitis C virus (HCV) disease progression in people who inject drugs (PWID): A systematic review and meta-analysis. *International Journal of Drug Policy*, *26*(10), 911-921.

Stone, J., Degenhardt, L., Grebely, J., Larney, S., Altice, F. L., Smyrnov, P., Rahimi-Movaghar, A., Alavi, M., Young, A. M., Havens, J. R., Miller, W. C., Hickman, M., & Vickerman, P. (2021). Modelling the intervention effect of opioid agonist treatment on multiple mortality outcomes in people who inject drugs: a three-setting analysis. *Lancet Psychiatry*, *8*(4), 301-309. <https://doi.org/10.1016/s2215-0366(20)30538-1>

Tseng, F. C., O'Brien, T. R., Zhang, M., Kral, A. H., Ortiz-Conde, B. A., Lorvick, J., Busch, M. P., & Edlin, B. R. (2007). Seroprevalence of hepatitis C virus and hepatitis B virus among San Francisco injection drug users, 1998 to 2000. *Hepatology*, *46*(3), 666-671. <http://www.ncbi.nlm.nih.gov/pubmed/17657818>
